# Unacceptably High Mortality Related to Measles Epidemics in Niger, Nigeria, and Chad

**DOI:** 10.1371/journal.pmed.0040016

**Published:** 2007-01-02

**Authors:** R. F Grais, C Dubray, S Gerstl, J. P Guthmann, A Djibo, K. D Nargaye, J Coker, K. P Alberti, A Cochet, C Ihekweazu, N Nathan, L Payne, K Porten, D Sauvageot, B Schimmer, F Fermon, M. E Burny, B. S Hersh, P. J Guerin

**Affiliations:** 1 Epicentre, Paris, France; 2 Ministère de la Santé et de la Lutte contre les Endémies du Niger, Niamey, Niger; 3 Ministère de la Santé Publique, N'Djamena, Chad; 4 Federal Ministry of Health, Abuja, Nigeria; 5 Cellule Interrégionale d'Epidémiologie d'Ile-de-France, Paris, France; 6 French Field Epidemiology Training Programme (PROFET), Institut de Veille Sanitaire, Saint-Maurice, France; 7 Health Protection Agency, Southwest Region, Stonehouse, Gloucestershire, United Kingdom; 8 European Programme for Intervention Epidemiology Training, Stockholm, Sweden; 9 Swedish Institute for Infectious Disease Control, Stockholm, Sweden; 10 Robert Koch-Institute, Berlin, Germany; 11 Norwegian Institute of Public Health, Oslo, Norway; 12 Médecins Sans Frontières, Paris, France; 13 Médecins Sans Frontières, Geneva, Switzerland; 14 World Health Organization, Geneva, Switzerland; World Health Organization, Switzerland

## Abstract

**Background:**

Despite the comprehensive World Health Organization (WHO)/United Nations Children's Fund (UNICEF) measles mortality–reduction strategy and the Measles Initiative, a partnership of international organizations supporting measles mortality reduction in Africa, certain high-burden countries continue to face recurrent epidemics. To our knowledge, few recent studies have documented measles mortality in sub-Saharan Africa. The objective of our study was to investigate measles mortality in three recent epidemics in Niamey (Niger), N'Djamena (Chad), and Adamawa State (Nigeria).

**Methods and Findings:**

We conducted three exhaustive household retrospective mortality surveys in one neighbourhood of each of the three affected areas: Boukoki, Niamey, Niger (April 2004, n = 26,795); Moursal, N'Djamena, Chad (June 2005, *n* = 21,812); and Dong District, Adamawa State, Nigeria (April 2005, *n* = 16,249), where n is the total surveyed population in each of the respective areas. Study populations included all persons resident for at least 2 wk prior to the study, a duration encompassing the measles incubation period. Heads of households provided information on measles cases, clinical outcomes up to 30 d after rash onset, and health-seeking behaviour during the epidemic. Measles cases and deaths were ascertained using standard WHO surveillance-case definitions. Our main outcome measures were measles attack rates (ARs) and case fatality ratios (CFRs) by age group, and descriptions of measles complications and health-seeking behaviour. Measles ARs were the highest in children under 5 y old (under 5 y): 17.1% in Boukoki, 17.2% in Moursal, and 24.3% in Dong District. CFRs in under 5-y-olds were 4.6%, 4.0%, and 10.8% in Boukoki, Moursal, and Dong District, respectively. In all sites, more than half of measles cases in children aged under 5 y experienced acute respiratory infection and/or diarrhoea in the 30 d following rash onset. Of measles cases, it was reported that 85.7% (979/1,142) of patients visited a health-care facility within 30 d after rash onset in Boukoki, 73.5% (519/706) in Moursal, and 52.8% (603/1,142) in Dong District.

**Conclusions:**

Children in these countries still face unacceptably high mortality from a completely preventable disease. While the successes of measles mortality–reduction strategies and progress observed in measles control in other countries of the region are laudable and evident, they should not overshadow the need for intensive efforts in countries that have just begun implementation of the WHO/UNICEF comprehensive strategy.

## Abstract 2

Three household retrospective mortality surveys in parts of West Africa affected by recent measles epidemics found that, despite progress made elsewhere, mortality rates remain unacceptably high.

## Editors' Summary

**Background.:**

In most developed countries, measles is often now regarded as an uncommon and not very serious childhood illness. The situation in developing countries is totally different; many children get measles, and the consequences can be severe. The main factor accounting for this difference is the much greater availability of vaccination against measles in developed countries. Globally, approximately 410,000 children under the age of 5 y die of measles each year. In developing countries, the death rate among children with measles is 1%–5%, but in refugee situations and among malnourished children, it may reach 10%–30%. The complications of the disease include pneumonia, diarrhea, encephalitis, and corneal scarring, which can lead to blindness. It costs less than US$1 to vaccinate a child against measles but, tragically, it remains the leading cause of vaccine-preventable death among children.

**Why Was This Study Done?:**

There are many national and international initiatives intended to improve measles vaccination rates, and in many developing countries things are improving; measles death rates in Africa as a whole are believed to be less than half of what they were 10 y ago. However, in certain countries—for example in West Africa—serious measles epidemics do still occur. It has been some years since any major studies have been conducted to try to establish how many children die during these epidemics. It is important to know this in order to help with efforts to improve the situation.

**What Did the Researchers Do and Find?:**

They focused on three epidemics of measles in West Africa and their impact on one neighborhood in each of three countries that were severely affected: Chad, Niger, and Nigeria. The total population of these neighborhoods was more than 64,000. The researchers spoke to the heads of households and asked for information about measles cases. They recorded details of symptoms of children who were taken ill during the epidemic and the outcome, including deaths. They also noted what action families took when children had measles. The percentage of children who developed measles was around 17% in the neighborhoods in Chad and Niger, and 24% in the Nigerian neighborhood. The death rate among the children who had measles was around 4% in Chad and Niger, and 11% in Nigeria. Most parents took their children to a health-care facility within 30 d of a rash appearing but this varied: 86% did so in Chad, 74% in Niger, and 53% in Nigeria.

**What Do These Findings Mean?:**

Children in these countries still face an unacceptably high risk of death from a completely preventable disease. Much more needs to be done to increase the number of children who are vaccinated.

**Additional Information.:**

Please access these Web sites via the online version of this summary at http://dx.doi.org/10.1371/journal.pmed.0040016

- Wikipedia information on measles (note that Wikipedia is an online encyclopedia that anyone can edit)
- More detailed information on measles may be obtained from MedlinePlus and the World Health Organization
- Information about the Measles Initiative
- For information about the three countries in this study, consult their country profiles on the BBC website: Chad, Niger, and Nigeria.

## Introduction

Very significant progress in measles control has been made over the past decade in Africa. The World Health Organization (WHO) and the United Nations Children's Fund (UNICEF) have developed a comprehensive strategy for sustainable measles mortality reduction, with the goal of a 90% reduction in global measles deaths (compared with 2000 levels) by 2010. The four-pronged strategy focuses on improved routine immunization, providing all children with a second opportunity for measles immunization through either periodic supplemental immunization activities (SIAs) or a routine second dose of measles vaccine, improved measles case management, and careful measles surveillance [1]. The launch of the Measles Initiative in 2001 has helped in the progress made. Partners in the initiative include: the American Red Cross, the United Nations Foundation, the Centers for Disease Control (United States), WHO, and UNICEF. In recent years, measles mortality has declined substantially in Africa: an estimated 216,000 measles deaths in the WHO African Region in 2004 compared with 519,000 in 1999 [2].

Despite this achievement, measles remains a leading cause of mortality in children under 5 y old in some sub-Saharan countries. Failure to deliver at least one dose of measles vaccine remains the primary reason for high measles mortality. Recurrent large epidemics continue in those countries that have not fully implemented the WHO/UNICEF comprehensive strategy. Measles epidemics generally occur in cycles, often with 1–3 y of relatively low incidence followed by an epidemic, commonly lasting 6 mo or more. The disease is a viral respiratory infection and affected children may suffer lifelong disabilities, including brain damage and blindness; fatalities result from complications including diarrhoea and pneumonia. To our knowledge, only a limited number of recent studies have been published on measles mortality during epidemics in high-burden African countries (see, for example, [3]), and recent reports focus on the success of the WHO/UNICEF measles mortality–reduction activities [4–9]. Here, we attempt to fill this information gap by examining measles morbidity and mortality in three epidemics in high-burden African countries.

Over the past several years, Epicentre/Médecins Sans Frontières (MSF) have been involved in the investigation of and response to measles epidemics in Niamey, Niger (2003–2004), Adamawa State, Nigeria (2004–2005), and N'Djamena, Chad (2004–2005). All three countries have chronically low immunization coverage and weak health systems; their populations are among the world's poorest; and they are identified as priorities for measles mortality reduction by the WHO/UNICEF strategic plan. All three countries have a routine measles vaccination schedule that aims to provide infants with a single dose at 9 mo of age, with no formal second opportunity for measles vaccination. Before the epidemics, SIA “catch-up” campaigns had yet to be conducted in any of these countries. Measles vaccination coverage in children aged under 5 y was estimated to be 33% in N'Djamena, Chad (Epicentre vaccination coverage survey, unpublished data); 70.9% in Niamey, Niger (Epicentre vaccination coverage survey [10]); and 22% (administrative data) in the north-eastern part of Nigeria where the epidemic in Adamawa State occurred [11].

At the beginning of each epidemic, at least ten cases were laboratory-confirmed by the respective Ministries of Health through detection of measles-specific IgM antibodies in sera collected after rash onset, following the WHO measles surveillance protocol [12]. Measles was diagnosed clinically using the standard WHO surveillance-case definition [13,14], and laboratory confirmation was not routinely performed.

MSF implemented outbreak-response immunization (Niamey and N'Djamena) and/or enhanced case management (Niamey, Adamawa State, and N'Djamena) in collaboration with the respective Ministries of Health. Vaccination activities were organized in Niamey and N'Djamena during the epidemics, taking place 24 wk and 22 wk after the beginning of the epidemic, respectively. A 1-wk campaign in Niamey targeted 50% of children aged between 6 mo and 59 mo living in the city, and in N'Djamena, a mass measles vaccination campaign targeted all children aged between 6 mo and 59 mo—both campaigns were implemented regardless of previous vaccination or disease history. Vaccine coverage subsequently reached 84.8% in Niamey [10] and 80.6% in N'Djamena (Epicentre, unpublished data). Reinforced clinical case management included training, provision of treatment kits comprising antibiotics, paracetamol, vitamin A, and oral rehydration salts. In all sites, strengthening of clinical case management, together with free access to treatment, was implemented at least 16 wk after the epidemic was detected (22 wk in Niamey, 18 wk in Adamawa State, and 16 wk in N'Djamena). In total, 10,880 cases were reported in Niamey, 8,015 cases in N'Djamena, and 2,505 cases in Adamawa State. Each of the three epidemics lasted for more than 6 mo.

We conducted exhaustive household retrospective mortality surveys in one neighbourhood of each of the three affected areas. The objective was to estimate measles age-specific attack rates (ARs) and case fatality ratios (CFRs), frequency of measles-related complications, and access to health care during these epidemics in order to provide a picture of measles morbidity and mortality in high-burden settings.

## Methods

With the agreement and collaboration of the respective Ministries of Health, Epicentre conducted three exhaustive household retrospective mortality surveys in Boukoki, Niamey (Niger); Moursal, N'Djamena (Chad); and Dong District, Demsa Local Government Area (Adamawa State, Nigeria). Niamey and N'Djamena are urban areas, whereas Demsa Local Government Area is a rural area.

Each neighbourhood covered a population of at least 16,000 persons according to the most recent population census. This population size was considered sufficient to investigate at least 500 measles cases, thereby allowing for an estimated measles CFR of 5% with a precision of ± 2.3% as described in the WHO protocol for determining measles CFRs in a community [15]. Site selection was based on rapid assessments carried out by field teams as well as discussions with health workers and community leaders. In addition to sufficient population size, clear administrative boundaries and feasibility issues (e.g., accessibility and the absence of major security concerns) were important selection criteria.

An epidemic was defined as an increase in measles cases during a 3-wk period above the number normally expected for the same period during non-epidemic years [13]. The recall period covered the time from the beginning of the epidemic until the start of the survey and was approximately 6 mo in all three sites (197 d in Boukoki, 184 d in Moursal, and 175 d in Dong District). The starting date coincided with the first day of Ramadan in Boukoki, the Ramadan mid-point in Dong District, and the first day of the year in Moursal. These dates provided an easily memorable event, and calendars of local events were used to facilitate date recall.

For all surveys, teams of interviewers visited every household in the study area. After completing the interview, the survey team left a mark on the house gate to ensure that all houses were visited and to avoid double counting. We asked for the help of neighbours to trace absentees and re-visited empty (but not abandoned) households later in the day. If during the second visit the occupants could not be found or if they refused to participate, that household was skipped. Interviewers conducted face-to-face interviews with heads of households, or, if they were absent, with the most senior adult household member. A household was defined as a group of people sleeping in the same house and sharing meals every day for at least the previous 2 wk. Oral, informed consent was obtained from all respondents before the interviews started. Standardized, pre-piloted questionnaires were used and interviews were conducted in local language(s).

For each household, we collected demographic data including sex and age (under 5 y, 5–14 y, and 15 y and over) at the end of the recall period and used this information as our denominator for the calculation of ARs. Measles cases were ascertained using the standard WHO clinical case definition: any person presenting with a history of fever and generalized macular-papular rash and at least one of coryza, cough, or conjunctivitis [13,14]. When measles cases were identified in a household, respondents provided information on age in months or years, sex, measles complications, disease outcome (dead or alive), and health-seeking behaviour. We considered two common complications, respiratory infections and diarrhoea, which were attributed to measles if they occurred within 30 d of rash onset. Respondents were asked whether children had a cough and/or breathing difficulties. Diarrhoeal complications were based on the WHO definition for diarrhoea [12].

For each death reported as being measles-related, we verified that the death met the following WHO definition: death in a person meeting the measles case definition within 30 d of rash onset, unless there was another clearly stated non-related cause [12]. Post-measles deaths occurring more than 30 d after rash onset were also estimated for the recall period. Place of death (house or health-care facility) was documented for each measles-associated death. It was not possible to corroborate deaths with death certificates, as declaration of deaths is not a common practice in the investigated populations.

Data were entered into EpiData version 3.0 (EpiData Association, http://www.epidata.dk) and analyzed using EpiInfo version 3.3 (CDC, http://www.cdc.gov). Measles ARs are expressed as measles cases per 100 persons, using the survey population as the denominator. Measles CFRs are expressed as measles deaths occurring within 30 d of rash onset per 100 cases, with a follow-up of at least 30 d. We also examined the CFRs for all measles-related deaths that occurred during the recall period, expressed as measles deaths occurring during the recall period per 100 cases, with a follow-up of at least 30 d.

## Results

**Figure 1:**
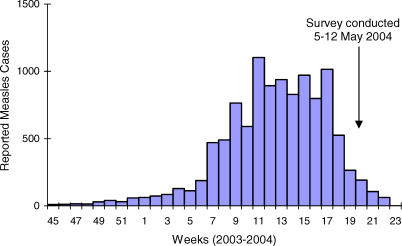
Reported Measles Cases in Niamey, Niger (2003–2004) (10,880 Patients)

**Figure 2:**
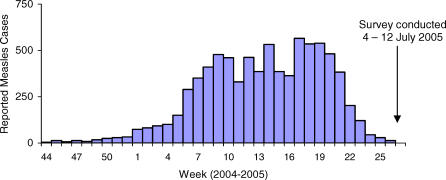
Reported Measles Cases, N'Djamena, Chad (2004–2005) (8,015 Patients)

**Figure 3:**
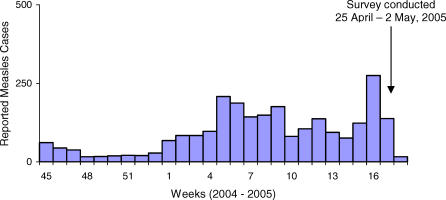
Reported Measles Cases, Adamawa State, Nigeria (2004–2005) (2,505 Patients)

Epidemic curves and the timing of the surveys are shown in Figures 1–3. Each survey took place when the epidemic was subsiding. Characteristics of study populations are representative of those expected in this region of Africa (Table 1). Fewer than 50 families refused in each survey, with the highest refusal rate being 2% in Dong District.

**Table 1:**
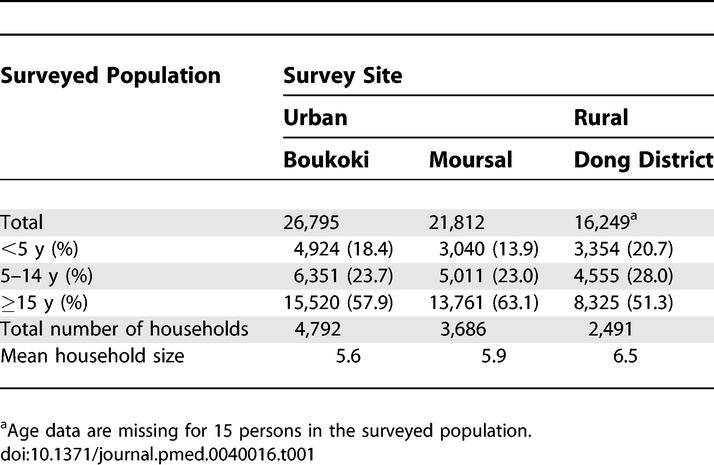
Description of Study Populations in Boukoki, Niamey, Niger; Moursal, N'Djamena, Chad; and Dong District, Adamawa State, Nigeria, 2003–2005

### Measles Morbidity

**Figure 4:**
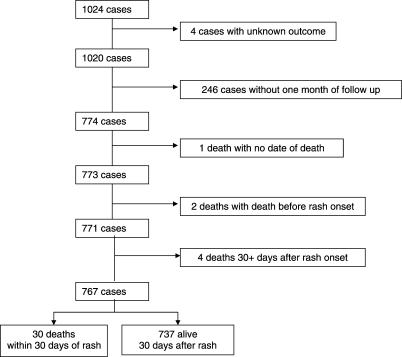
Flow Diagram of Measles Cases Included in the Study, Boukoki, Niamey, Niger (2003–2004)

**Figure 5:**
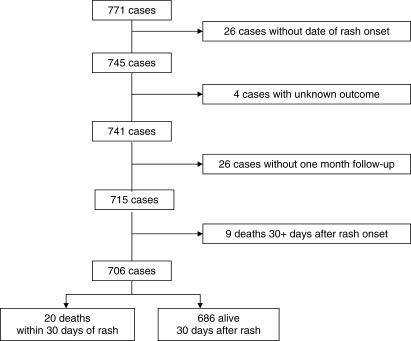
Flow Diagram of Measles Cases Included in the Study, Moursal, N'Djamena, Chad (2004–2005)

**Figure 6:**
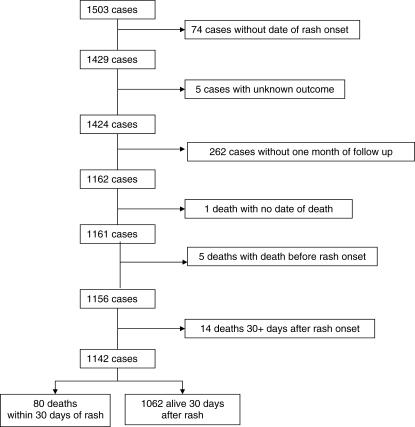
Flow Diagram of Measles Cases Included in the Study, Dong District, Adamawa State, Nigeria (2004–2005)

During the surveyed period, we counted 1,024 measles cases in Boukoki (AR = 3.8%), 745 in Moursal (3.4%), and 1,429 in Dong District (8.8%) (Figures 4–6; Table 2). The highest ARs were found in children aged under 5 y (Table 2).

**Table 2:**
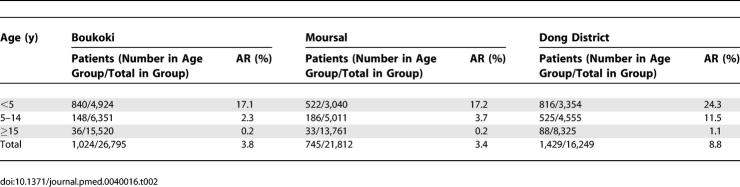
Age-Specific Measles ARs in Boukoki, Niamey, Niger; Moursal, N'Djamena, Chad; and Dong District, Adamawa State, Nigeria, 2003–2005

In Boukoki, previous measles vaccination (confirmed by card) was reported for 37.3% (382/1,024) of patients, with 70% (522/745) reported in Moursal. In Dong District, only 1.0% (15/1,429) of patients had documented measles vaccination. Few cases were in children aged less than 9 mo (the age of routine vaccination) in each of the three sites: 5.5% (56/1,024) in Boukoki, 5.6% (42/745) in Moursal, and 4.1% (58/1,429) in Dong District.

Of measles cases with information on complications, 65.3% (506/775) of patients in Boukoki, 79.0% (588/744) of patients in Moursal, and 67.0% (770/1,149) of patients in Dong District developed respiratory complications; diarrhoeal complications were reported for 60.1% of patients (466/775) in Boukoki, 71.7% (521/727) of patients in Moursal, and 66.3% (762/1,149) of patients in Dong District.

### Measles Mortality

The median time between rash onset and death was 9 d in Boukoki, 6 d in Moursal, and 11 d in Dong District. The median age of death was 18 mo in Boukoki (interquartile range [IQR]: 11–33 mo), 19 mo in Moursal (IQR: 10–26 mo), and 24 mo in Dong District (IQR: 24–36 mo).

For deaths occurring within 30 d of rash onset among patients with at least 30 d of follow-up, the highest measles CFR occurred in all sites in children aged less than 5 y (Table 3). When children aged under 5 y were disaggregated into finer age ranges, CFR declined with age except in Dong District, where the CFR in 12–35-mo-old children was higher than that in those under 12 mo (Table 4).

**Table 3:**
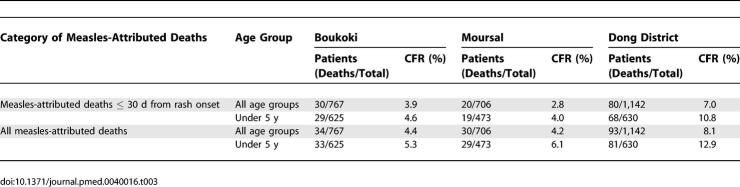
Measles CFRs in Boukoki, Niamey, Niger; Moursal, N'Djamena, Chad; and Dong District, Adamawa State, Nigeria, 2003–2005

**Table 4:**
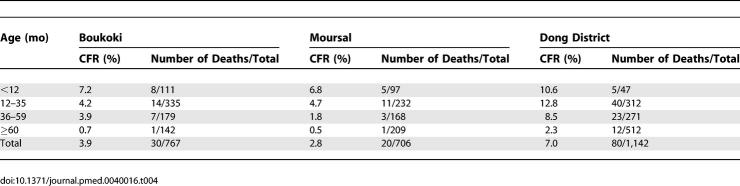
Age-Specific Measles CFR in Boukoki, Niamey, Niger; Moursal, N'Djamena, Chad; and Dong District, Adamawa State, Nigeria, 2003–2005

Between 30 and 60 d of rash onset, a total of three deaths (8.8%) occurred in Boukoki, with six deaths (20.7%) in Moursal and seven deaths (7.4%) in Dong District. Few deaths occurred after 60 d of rash onset: one death (2.9%) in Boukoki, three deaths (10.3%) in Moursal, and six deaths (6.4%) in Dong District.

Of the 4,792 households visited in Boukoki, 30 reported a measles death, and of the 816 households reporting measles cases, 3.4% (28/816) reported at least one death. Of all households visited in Moursal, 0.5% (17/2,686) reported a measles death, and of the 533 households reporting measles cases, 3.2% reported at least one death (17/533). One or more measles deaths occurred in 2.9% (71/2,491) of all households visited in Dong District and in 10.3% (71/690) of all households with measles cases in Dong District.

One measles death occurred in Boukoki with card-confirmed measles vaccination, two deaths in Moursal, and one death in Dong District. Considering parental recall of vaccination, three deaths occurred in vaccinated individuals in Boukoki, ten deaths in Moursal, and five deaths in Dong District. Approximately half of the deaths in both Boukoki (16/30) and in Moursal (10/20) reported not having taken Vitamin A in the 6 mo prior to rash onset or in the month after rash onset. In Dong District, 62.5% (50/80) reported not having taken Vitamin A in the 6 mo prior to rash onset or in the month after rash onset.

There were no reported differences between deaths in males and females in Boukoki (male-to-female ratio [M:F ratio] 1.3:1 [17:13, *p* = 0.79]) or in Moursal (M:F ratio 1.5:1 [12:8, *p* = 0.75]). In Dong District, a larger proportion of deaths occurred among females than among males (M:F ratio 0.4:1 [21:59, *p* < 0.001]), while the M:F ratio in the measles case population was 1.0.

During our surveys, we also recorded data on all deaths that occurred during the recall period (results not shown). In all sites, 50% of all deaths in children aged under 5 y were attributed to measles. The under-5-y mortality rate in Boukoki was 0.8 deaths/10,000 persons/d, and in Moursal the under-5-y mortality rate was 1.0 deaths/10,000 persons/d. In Dong District, the under-5-y mortality rate was 2.8 deaths/10,000 persons/d, which is above the conventional threshold of 2.0 deaths/10,000 persons/d used to characterize a complex humanitarian emergency.

### Access to Health Care

Of measles cases, it was reported that 85.7% (979/1,142) of patients visited a health-care facility within 30 d after rash onset in Boukoki, with 73.5% (519/706) in Moursal and 52.8% (603/1,142) in Dong District. Of patients who consulted a health-care facility, 95.0% (930) in Boukoki, 85.2% (442) in Moursal, and 95.2% (574) in Dong District paid for services. Little information on measles CFR in health-care facilities was available. In Niamey General Hospital (Boukoki) and Numan General Hospital (Dong District), the main health-care facilities to which patients with measles complications were admitted or referred, 19.0% and 12.9% of patients, respectively, died during the epidemics. This information was not available for the epidemic in N'Djamena.

Almost two-thirds (19/30, 63.3%) of deaths attributed to measles occurred at home in Boukoki and more than half in Moursal (57.1%, 12/21). Of the 80 deaths in Dong District, 70 occurred at home (87.5%). Of deaths occurring at home in any of the study sites, none of the patients visited a medical facility, and the principal reasons were different in each of the study sites. In Boukoki, the principal reasons provided for not visiting a medical facility were lack of money (62%) or that the patient was not seriously ill (11%). In both Moursal and Dong District, the principal reasons given were that the patient was treated at home with treatment purchased from a pharmaceutical vendor or market (49% in both locations); that traditional medicine was used (27% in Moursal and 19% in Dong District); and that the patient did not have the money to pay for the visit (11% in Moursal and 16% in Dong District).

## Discussion

Our results provide recent estimates from a region of sub-Saharan Africa that has not experienced the progress in measles control observed in other countries of the region. The 2.8%–7.0% CFRs found in our study are as high as those reported in the early 1990s [3,16–20] from these same countries that were included in our surveys. Similar retrospective community studies conducted in Boukoki, Niamey, Niger after measles epidemics estimated an overall CFR of 6.6% in 1991 [16] and a CFR of 2.4% in children aged under 5 y in 1995 [17]. In a rural area in Niger, a retrospective community survey that was performed after the 1991–1992 epidemic identified a CFR of 18.2% in children aged under 5 y [18]. A recent study in Mirriah district in Niger found a CFR of 9.7% [3]. In N'Djamena, a community survey after a measles epidemic in 1993 found a mean CFR of 7.4% in children aged under 5 y [20]. In an urban area of Nigeria in 1995, a CFR of 3.3% was found in the population [19].

The continuing high burden of preventable measles mortality during these epidemics results from poor access to appropriate treatment and the incomplete implementation of the WHO/UNICEF measles mortality–reduction strategy. In all sites, access to appropriate treatment was not provided free of charge from the beginning of the epidemic. In the rural site of Dong District, where the measles CFR was highest, less than half of all measles patients had contact with a health-care facility, and in all sites, most measles deaths occurred at home. Respondents often preferred to purchase treatment outside of a health-care facility (e.g., market or pharmaceutical vendor), rather than to seek treatment at a health-care facility. Moreover, in health-care facilities admitting patients with more severe measles complications, excess workload and inappropriate case management of measles complications may have contributed to the high measles mortality observed in the principal referral hospitals in Niamey and Dong District.

The high mortality seen in these three epidemics also resulted from incomplete implementation of the recommended strategy for sustainable measles mortality reduction [1]. Routine vaccination programs in these countries have not been able to consistently provide a high proportion of infants and young children with measles vaccine through routine health services. Moreover, vaccination programs in these countries make limited efforts to reach older children who failed to receive measles vaccine through routine services. The absence of recent SIAs, together with chronically low vaccine coverage, have combined to allow the numbers of measles-susceptible children to build up to very high levels and to precipitate these epidemics. Furthermore, outbreak-response vaccination activities occurred very late in the epidemic in Niamey and N'Djamena and not at all in Dong District. It is important to note that SIAs targeting all children of between 9 mo and 14 y have been implemented in all three study sites since the completion of our surveys: in December 2004 in Niger (99% estimated coverage obtained as a percentage of target population), and in December 2005 in both Nigeria (96%) and Chad (80%). There were no measles epidemics reported in 2006, although we would not expect an epidemic immediately after a high-coverage SIA or a major epidemic.

SIAs may be very effective in quickly reducing measles mortality [21], but the impact can be sustained only if they are a component of a comprehensive and long-term measles-immunization strategy. If national governments do not have the ability to sustain implementation of a comprehensive strategy, SIAs may result only in a short-term reduction of measles deaths. In large countries with poor public health infrastructures and high birth rates, which is the situation in the locations investigated here, there are major logistical and financial challenges for governments to overcome. At the same time, these constraints are only partially responsible for the high measles burden and should not be used as arguments not to improve control measures.

Although ARs were similar in all three sites, the CFR was higher in Dong District. This is likely due to several factors. First, there was no vaccination intervention and, for half of reported cases, respondents reported no contact with a medical facility. Second, MSF and the Ministry of Health implemented free access to treatment for patients with measles in April 2005, 6 mo after the start of the epidemic. Consequently, for most patients included in the survey who consulted a health-care facility, the treatment was paid for, with an average of 6 euros per consultation. After free treatment was provided, measles case attendance increased, but it was observed that most cases continued to come from areas adjacent to health-care facilities. This geographic limitation of rural areas was not observed in the two urban areas investigated here.

There were several limitations to these studies. Measles cases that were ascertained during the community surveys were not laboratory-confirmed, except for ten to 20 cases at the beginning of each of the outbreaks. This may have resulted in a classification bias resulting in an overestimation of measles AR and CFRs. In all study sites, measles is a well-known disease entity. Thus, we would expect the accuracy of respondents reporting cause of deaths to be high [22], and the positive predictive value of a case satisfying the clinical case definition would also be expected to be high.

As these studies were conducted towards the end of the epidemics, respondents might have attributed unrelated deaths to measles. Conversely, we cannot exclude the possibility that, owing to the long delay between the disease and the survey, measles infections that occurred at the beginning of the recall period were not reported. In all three sites, the majority of measles deaths reported occurred in the second half of the study period, i.e., during the months of February, March, and April (73% in Dong District, 70% in Boukoki, and 79% in Moursal). Furthermore, the denominator used to estimate measles ARs was the study population, and not the number of persons susceptible to measles, thereby underestimating ARs.

Our study-site selection may also not be representative of the affected areas. Site selection was based on population size and logistic considerations, thereby favouring areas that are more accessible. As a result, these areas likely had health-care facilities capable of detecting measles cases through surveillance and therefore may not be representative of the area as a whole. We also cannot exclude the possibility that survey teams may have missed households in the study areas. However, as survey teams were trained extensively and returned to households where the head of household was absent, this is likely to be a very small proportion. An additional limitation concerns the difficulty in ascertaining age in the populations surveyed. Births and deaths are not routinely recorded, and the age of children is almost always imprecise. As these surveys were conducted in only one neighbourhood of each of the affected areas, our results may be suitable for extrapolation only to other similar contexts.

We considered only respiratory infections and diarrhoea as measles complications. For other known complications, we could not ensure the accuracy of respondents as case definitions were complex and could be easily misinterpreted (e.g., corneal ulcer versus conjunctivitis, encephalitis versus septic shock). Other complications, for example malnutrition, were likely to be present in these study populations. Furthermore, some studies have suggested that mortality related to measles may be delayed for as long as 1 y after acute diseases have been observed [23]. In our populations, only a small proportion occurred after the 30-d window, but respondents may not have attributed a death occurring more than 30 d after rash onset to measles.

Measles deaths, unlike some other high-burden diseases in sub-Saharan Africa (tuberculosis, malaria, and HIV/AIDS), can be fully prevented by using an effective, safe, and cheap vaccine under proven vaccination strategies. Governments, international non-governmental organizations, and international partnerships like the Measles Initiative should strengthen their efforts in those countries where measles continues to kill thousands of children every year. Recognizing shortcomings in measles vaccination and mortality-reduction programs could be the first step towards eliminating measles in Africa [24].

## Supporting Information

Alternative Language Abstract S1 Translation of the Abstract into French by R. F. Grais (21 KB DOC)

Alternative Language Abstract S2 Translation of the Abstract into Spanish by R. F. Grais (23 KB DOC)

## Abbreviations

AR: attack rate
CFR: case fatality ratio
IQR: interquartile range
M:F ratio: male-to-female ratio
MSF: Médecins Sans Frontières
SIA: supplemental immunization activity
UNICEF: United Nations Children's Fund
WHO: World Health Organization
