# Supplementary material for: Unacceptably High Mortality Related to Measles Epidemics in Niger, Nigeria, and Chad
Source: PLoS Med. 2007 Jan 2;4(1):e16. doi: 10.1371/journal.pmed.0040016 (PMC1761051; doi:10.1371/journal.pmed.0040016)
Supplement: Supplementary file 1 [file pmed.0040016.sd001.doc]

**Summary**

# Background:

Malgré la stratégie conjointe de réduction de la mortalité de la rougeole de l’OMS/UNICEF et de l’Initiative Rougeole (Measles Initiative) en un partenariat d'aide à la réduction de la mortalité liée à la rougeole en Afrique, certains pays à haut risque continuent à faire face à des épidémies récurrentes. Peu d'études récentes ont documenté la mortalité de la rougeole en Afrique sub-saharienne. L'objectif était d'étudier la mortalité de la rougeole lors de trois épidémies récentes à Niamey (Niger), N'Djamena (Tchad) et dans l’état d'Adamawa (Nigéria).

**Méthodes :** Nous avons effectué trois enquêtes de mortalité rétrospective basées sur un interrogatoire des chefs de famille des ménages vivant dans le voisinage de chacun des trois sites atteints: Boukoki, Niamey, Niger (avril 2004, n=26795) ; Moursal, N'Djamena, Tchad (juin 2005, n=21812) ; et la zone de Dong, Etat d'Adamawa, Nigéria (avril 2005, n=16249). Les populations d'étude comprenaient tous les habitants résidant sur les sites durant les deux semaines précédant l'étude, une durée qui couvrait la période d'incubation de la rougeole. Les chefs de famille ont fourni des informations sur les cas de rougeole, les évènements cliniques dans les 30 jours suivant l’éruption, et d’éventuelles consultations au centre de santé pendant l'épidémie. Les définitions standard de l’OMS des cas et des décès dus à la rougeole ont été utilisées. Nous présentons les résultats des taux d'attaque de la rougeole (AR), des taux de mortalité (CFR) par groupe d'âge, ainsi que la description des complications de la rougeole et des éventuelles consultations au centre de santé.

**Principaux résultats :** Le taux d’attaque de la rougeole était le plus important chez les enfants de moins de 5 ans: 17,1 % à Boukoki, 17,2% à Moursal et 24,3% dans la zone de Dong. Les CFRs des moins de 5 ans étaient de 4,6%, 4,0% et 10,8% dans les zones de Boukoki, Moursal et Dong respectivement. Dans tous les sites, pour plus que la moitié des cas de rougeole des moins de 5 ans, il a été reporté une infection respiratoire et/ou une diarrhée aiguë dans les 30 jours suivant le début de l’éruption. Parmi des cas de rougeole, il a été mentionné une consultation dans un centre de santé dans les 30 jours suivant le début de l’éruption dans 85,7% (979/1142) des cas à Boukoki, 73,5% (519/706) à Moursal et 52,8% (603/1142) dans la zone de Dong.

**Conclusion :** La mortalité infantile due à la rougeole, maladie complètement évitable, dans ces pays reste très importante. Bien que les succès des stratégies de réduction de mortalité de rougeole soient louables et évidents, ils ne doivent pas éclipser le besoin d'efforts intensifs dans les pays qui viennent de débuter ces stratégies.
