# Supplementary material for: Unacceptably High Mortality Related to Measles Epidemics in Niger, Nigeria, and Chad
Source: PLoS Med. 2007 Jan 2;4(1):e16. doi: 10.1371/journal.pmed.0040016 (PMC1761051; doi:10.1371/journal.pmed.0040016)
Supplement: Supplementary file 2 [file pmed.0040016.sd002.doc]

**Resumen**

# Contexto:

A pesar de la estrategia integrada para la reducción de la mortalidad por el sarampión preconizada por la OMS/UNICEF y la “Measles Initiative” (una colaboración en apoyo a la reducción de la mortalidad por sarampión en Africa), ciertos países fuertemente afectados continúan haciendo frente a epidemias recurrentes. Pocas encuestas recientes han estudiado la mortalidad por sarampión en Africa sub-sahariana. Nuestro objetivo fue determinar la mortalidad por sarampión en tres epidemias recientes en Niamey (Niger), N'Djamena (Chad) y el estado de Adamawa (Nigeria).

**Métodos:** Realizamos tres encuestas retrospectivas de mortalidad en un municipio de cada una de las tres áreas afectadas: Boukoki (Niamey, Niger; abril 2004, n=26.795); Moursal, (N'Djamena, Chad; junio 2005, n = 21.812); y el distrito de Dong (estado de Adamawa, Nigeria; abril 2005, n=16.249). El estudio incluyó todos los residentes de al menos dos semanas de antigüedad, abarcando el tiempo de incubación del sarampión. Los jefes de familia proporcionaron la información sobre los casos de sarampión, resultados clínicos en los 30 días siguientes al inicio de la erupción, y de búsqueda de atención médica durante la epidemia. Los casos y las muertes por sarampión fueron clasificados usando definiciones estándard de la OMS. Los indicadores de evaluación empleados fueron las tasas de ataque (TA) y la fatalidad atribuida (CFR) al sarampión por grupo etario, descripción de las complicaciones del sarampión y de búsqueda de atención médica durante la epidemia**.**

**Resultados principales:** Los niños menores de 5 años mostraron las TA más elevadas: 17,1% en Boukoki, 17,2% en Moursal y 24,3% en el distrito de Dong. Los CFRs en el mismo grupo etario fueron 4,6%, 4,0% y 10,8% en Boukoki, Moursal y Dong respectivamente. En los tres sitios, más de la mitad de los casos de sarampión en menores de 5 años sufrieron infecciones respiratorias y/o diarrea agudas durante los 30 días siguientes al inicio de la erupción. De las personas afectadas de sarampión, 85,7% (979/1.142) declararon acudir a un centro de salud durante los 30 días siguentes al inicio de la erupción en Boukoki, 73,5% (519/706) en Moursal y 52,8% (603/1.142) en el distrito de Dong.

**Conclusiones:** En estos países, la mortalidad infantil debida a enfermedades prevenibles es inaceptablemente elevada. El progreso alcanzado por las estrategias de reducción de la mortalidad por sarampión en otros países de la región, no debería ocultar la necesidad de intensificar los esfuerzos para desarrollar la estrategia integrada preconizada por la OMS/UNICEF en países que iniciaron su implementación recientemente.
